# Cell death programs in *Yersinia* immunity and pathogenesis

**DOI:** 10.3389/fcimb.2012.00149

**Published:** 2012-11-30

**Authors:** Naomi H. Philip, Igor E. Brodsky

**Affiliations:** ^1^Immunology Graduate Group, School of Veterinary Medicine, University of PennsylvaniaPhiladelphia, PA, USA; ^2^Department of Pathobiology, University of PennsylvaniaPhiladelphia, PA, USA; ^3^Institute for Immunology, University of PennsylvaniaPhiladelphia, PA, USA

**Keywords:** *Yersinia*, cell death, YopJ, inflammasome, caspase-1, apoptosis, pyroptosis

## Abstract

Cell death plays a central role in host-pathogen interactions, as it can eliminate the pathogen's replicative niche and provide pro-inflammatory signals necessary for an effective immune response; conversely, cell death can allow pathogens to eliminate immune cells and evade anti-microbial effector mechanisms. In response to developmental signals or cell-intrinsic stresses, the executioner caspases-3 and -7 mediate apoptotic cell death, which is generally viewed as immunologically silent or immunosuppressive. A proinflammatory form of cell death that requires caspase-1, termed pyroptosis, is activated in response to microbial products within the host cytosol or disruption of cellular membranes by microbial pathogens. Infection by the bacterial pathogen *Yersinia* has features of both apoptosis and pyroptosis. Cell death and caspase-1 processing in *Yersinia*-infected cells occur in response to inhibition of NF-κB and MAPK signaling by the *Yersinia* virulence factor YopJ. However, the molecular basis of YopJ-induced cell death, and the role of different death pathways in anti-*Yersinia* immune responses remain enigmatic. Here, we discuss the role that cell death may play in inducing specific pro-inflammatory signals that shape innate and adaptive immune responses against *Yersinia* infection.

## Introduction

Cell death plays a key role in maintaining tissue homeostasis by eliminating stressed, damaged, and infected cells. Cell death is an evolutionarily conserved immune response to microbial infection, as it prevents pathogen replication and can provide pro-inflammatory signals necessary for an effective immune response. Distinct cell death pathways that result in distinct downstream outcomes are induced under different circumstances (Kono and Rock, 2008; Green et al., 2009; Zitvogel et al., 2010). Apoptosis is traditionally viewed as an immunomodulatory form of cell death characterized by cell shrinkage, while necrosis and pyroptosis are pro-inflammatory forms of death associated with rapid loss of membrane integrity and release of intracellular contents. How these distinct cell death pathways contribute to antibacterial responses remains an important unanswered question. Recent studies indicate that caspase-1-dependent pyroptosis can promote antibacterial responses against intracellular pathogens independent of production of the caspase-1 dependent cytokines IL-1β and IL-18 (Miao et al., 2010). Similarly, RIP3-dependent necrosis, a recently described form of programmed necrosis (Cho et al., 2009; He et al., 2009; Zhang et al., 2009; Oberst et al., 2011), promotes control of *Vaccinia* and CMV viral infections (Upton et al., 2010). Both pyroptosis and programmed necrosis can occur in the context of pathological conditions that are not directly associated with microbial infections (Martinon et al., 2006; Welz et al., 2011; Inoue et al., 2012). However, how different cell death pathways contribute to cytokine production, and orchestrate activation of innate and adaptive cells during bacterial infections remains a major unresolved question for understanding of anti-*Yersinia* immunity.

The three pathogenic *Yersinia* spp., *Y. pestis, Y. pseudotuberculosis*, and *Y. enterocolitica*, share a virulence plasmid encoding a conserved Type Three Secretion System (T3SS) and virulence factors, known as *Yersinia* outer proteins (Yops) (Viboud and Bliska, 2005). T3SS-mediated injection of Yops into infected cells enables *Yersinia* to modulate host signaling pathways and suppress innate and adaptive immunity (Cornelis, 2006). YopJ of *Y. pestis* and *Y. pseudotuberculosis*, termed YopP in *Y. enterocolitica*, blocks NF-κ B and MAPK signaling, thereby inhibiting cytokine production and triggering death of *Yersinia* infected cells (Mills et al., 1997; Monack et al., 1997; Ruckdeschel et al., 1998). Among the sequenced strains of pathogenic *Yersiniae*, YopJ and YopP share 95–98% identity across the full length of the protein sequence, but key polymorphisms have been identified that impact both enzymatic activity and translocation of the protein, which affect the outcome of *Yersinia* infection (Ruckdeschel et al., 2001b; Zauberman et al., 2006; Brodsky and Medzhitov, 2008; Zheng et al., 2011).

Interestingly, *Yersinia*-infected cells exhibit features of apoptosis, pyroptosis, or necrosis, depending on the state of the cells and the cell type involved (Monack et al., 1997; Ruckdeschel et al., 1997, 1998, 2001a; Bergsbaken and Cookson, 2007; Zheng et al., 2012). However, whether YopJ-induced cell death promotes host defense or bacterial virulence during *in vivo* infection remains unclear. A number of studies have revealed key players in cell death pathways during *Yersinia* infection, providing some insight into mechanisms of *Yersinia*-induced cell death, but key questions about the nature and role of *Yersinia* death *in vivo* remain. *Yersinia* is thought to primarily replicate as an extracellular pathogen that evades phagocytosis by neutrophils and monocytic cells in lymphoid tissues. Cell death has therefore been viewed as a strategy for *Yersinia* to eliminate host phagocytes (Monack et al., 1998). However, several studies suggest that host cell death during *Yersinia* infection may promote anti-*Yersinia* immunity, although the precise mechanisms are not entirely clear (Brodsky and Medzhitov, 2008; Bergman et al., 2009; Zauberman et al., 2009). An alternative possibility is that the *Yersiniae* are capable of intracellular replication, suggesting the existence of an intracellular stage during the *Yersinia* lifecycle *in vivo* (Grabenstein et al., 2004).

## Mechanisms of *Yersinia*-induced cell death

Early studies observed that macrophages and dendritic cells infected by *Yersinia* exhibit characteristics of apoptotic cells, specifically membrane blebbing, nuclear condensation, DNA fragmentation, and formation of large cytoplasmic vacuoles (Monack et al., 1997; Ruckdeschel et al., 1997). Apoptosis has been viewed as immunologically silent, but growing evidence suggests that during infection, apoptosis may promote inflammatory responses (Green et al., 2009; Torchinsky et al., 2009). Furthermore, apoptotic cells can be phagocytosed, and their associated microbial antigens used to prime CD8^+^ T cell responses (Heath and Carbone, 2001). Therefore, while cell death during *Yersinia* infection is thought to be apoptotic, it may not be immunologically silent. Below, we discuss the nature of *Yersinia*-induced cell death and its contribution to bacterial virulence or host defense.

**Figure 1:**
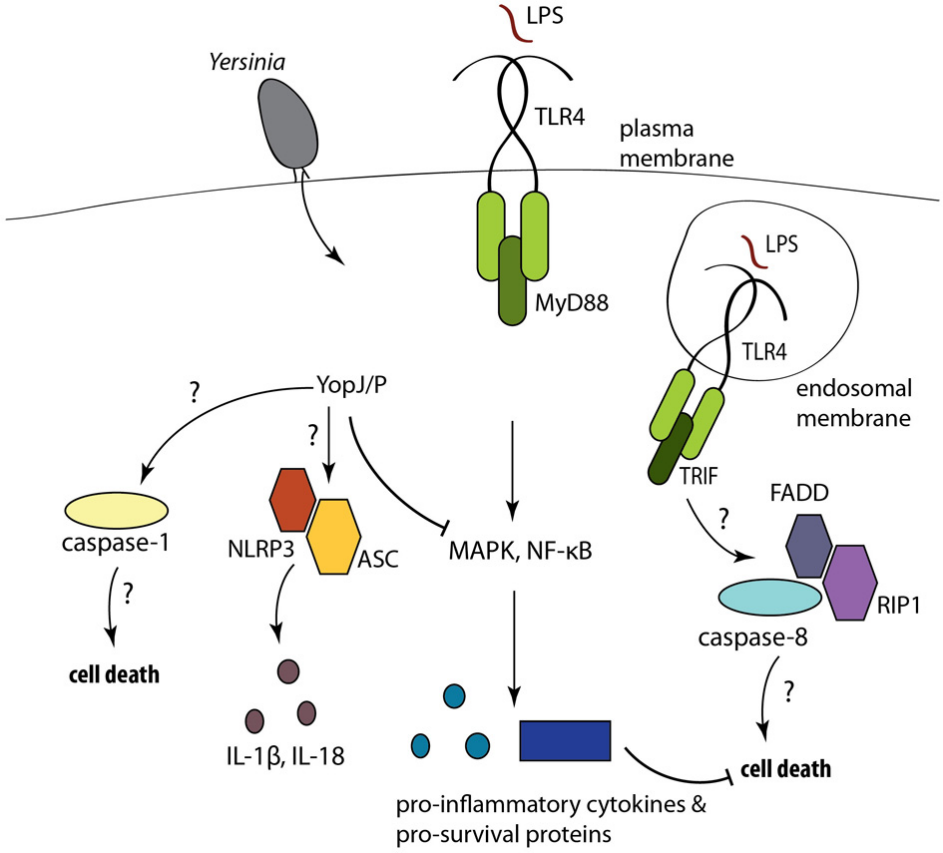
**Yops are injected into phagocytes during *Yersinia* infection.** YopJ/P inhibits NF-κ B and MAPK signaling, preventing the expression of pro-inflammatory cytokines and pro-survival molecules, resulting in cell death. YopJ also activates caspase-1 processing and IL-1β and IL-18 release. The mechanisms of cell death and inflammasome activation are not well-understood.

The cysteine protease YopJ, called YopP in *Y. enterocolitica*, induces cell death during *Yersinia* infection (Mills et al., 1997; Monack et al., 1997, 1998). YopJ is a potent inhibitor of MAPK and NF-κ B signaling, and blocks proinflammatory cytokine production by infected cells (Ruckdeschel et al., 1998, 2001a; Palmer et al., 1999; Orth et al., 2000) (Figure 1). YopJ has been reported to function as an ubiquitin-like protein protease (Orth et al., 2000), and as a deubiquitinase (Zhou et al., 2005; Sweet et al., 2007). YopJ is also reported to be an acyl transferase that acetylates serine residues in the activation loop of MKK family proteins and prevents their activation (Mittal et al., 2006; Mukherjee et al., 2006). A recent study has also indicated that the sensitivity of NF-κ B signaling pathways to YopJ-mediated inhibition occurs at the level of TAK1 and is evolutionarily conserved from *Drosophila* to mammalian cells (Paquette et al., 2012).

Macrophages stimulated with LPS in the presence of inhibitors of protein synthesis or components of NF-κB signaling also undergo cell death (Ruckdeschel et al., 2004; Zhang et al., 2005). Consistent with this, *Tlr4^−/−^* macrophages are resistant to YopJ-dependent apoptosis, as are cells deficient in the TLR3/4 adaptor TRIF, but not MyD88 (Haase et al., 2003; Zhang and Bliska, 2003; Ruckdeschel et al., 2004). Moreover, infection of dendritic cells with *Yersinia* leads to the formation of a FADD/caspase-8/RIP1 complex and caspase-8 activation (Figure 1) (Grobner et al., 2007). Cytochrome-c release and caspase-9 cleavage were observed downstream of *Yersinia*-induced cleavage of the pro-apoptotic Bid protein (Denecker et al., 2001). Additionally, treatment with broad-spectrum caspase inhibitors reduced the number of TUNEL^+^ cells during *Yersinia* infection (Monack et al., 1998; Denecker et al., 2001). Collectively, these data indicate that *Yersinia* activates an extrinsic pathway of apoptosis. Interestingly, *Yersinia* infection of dendritic cells exposed to a pan-caspase inhibitor still induced a cell death that is presumably caspase-independent and exhibited morphological features of necrosis (Grobner et al., 2006). Recent studies have described a caspase-independent pathway of programmed necrosis linked to TRIF that involves signaling through the RIP1 and RIP3 kinases (Vandenabeele et al., 2010; Feoktistova et al., 2011; Tenev et al., 2011), but how this pathway functions during *Yersinia* infection and the link between programmed necrosis and other forms of *Yersinia*-induced death is not known.

Pathogens express pore-forming toxins and virulence proteins that disrupt membrane integrity and modulate signaling networks (Vance et al., 2009). Consequently, mammalian hosts have evolved mechanisms, including the activation of a multiprotein complex called the inflammasome, that detect these virulence activities (Schroder and Tschopp, 2010). Inflammasomes are activated by Nod-like Receptors (NLRs) that contain a leucine-rich-repeat (LRR) sensor domain, a nucleotide-binding oligomerization domain (NOD) and a pyrin- or CARD-containing signaling domain (Davis et al., 2011). Inflammasome activation is thought to require two signals, the first one involving sensing of conserved PAMPs that induce expression of certain inflammasome components and cytokines, and a second signal involving disruption of cellular membranes or signaling pathways (Mariathasan and Monack, 2007). Inflammasomes form a platform for the autoprocessing and activation of the cysteine protease caspase-1, resulting in caspase-1-dependent secretion of IL-1α, IL-1β, and IL-18, and caspase-1-dependent cell death termed pyroptosis (Mariathasan et al., 2004; Martinon et al., 2006; Sutterwala et al., 2006; Bergsbaken et al., 2009; Hornung et al., 2009). *Yersinia* expresses a conserved T3SS that activates the NLRP3 and NLRC4 inflammasomes, triggering pyroptosis (Brodsky et al., 2010). However, the virulence factor YopK prevents this inflammasome activation, and promotes bacterial replication and dissemination *in vivo* (Brodsky et al., 2010). In the presence of YopJ, YopK-sufficient bacteria still induce cell death. Thus, a key question is how these two seemingly contradictory outcomes are controlled during *Yersinia* infection. *Yersinia* expressing YopK but lacking YopJ, do not induce T3SS-dependent inflammasome activation or cell death. Thus, YopK likely limits inflammasome activation under conditions where YopJ expression or translocation is reduced, as may happen during infection of systemic sites (discussed further below). Nevertheless, macrophages primed by inflammatory stimuli still undergo pyroptosis in response to YopJ-deficient *Yersinia* (Bergsbaken and Cookson, 2007), which is greatly enhanced in the additional absence of YopK (Brodsky et al., 2010). As LPS priming upregulates NLRP3 inflammasome components (Bauernfeind et al., 2009), the threshold for NLRP3 inflammasome activation could be lowered, even in the presence of YopK.

Interestingly, YopJ-dependent apoptosis is also associated with caspase-1 activation (Lilo et al., 2008; Brodsky et al., 2010; Zheng et al., 2011), and the extent of YopJ-mediated NF-κ B inhibition correlates with the degree of caspase-1 activation (Zheng et al., 2011), consistent with the finding that deletion of IKKβ in macrophages induces spontaneous inflammasome activation (Greten et al., 2007). Although NLRP3 and the adaptor ASC are required for YopJ-dependent secretion of IL-1β and IL-18 (Zheng et al., 2011), the mechanism by which YopJ activates caspase-1 is unclear, as caspase-1 processing and YopJ-dependent cell death still occur in cells lacking ASC, NLRC4, or NLRP3 (Brodsky et al., 2010). Distinct inflammasome complexes with different functions have been identified, and could potentially account for these observations (Figure 1). Caspase-1 could be recruited to an NLRP3/ASC complex that regulates IL-1β and IL-18 production, and to a separate complex that activates cell death. A complex containing catalytically active caspase-1, but not ASC triggers cell death but not cytokine secretion, while a distinct ASC-containing focus mediates caspase-1 processing and cytokine secretion, during *Salmonella* infection (Broz et al., 2010). NLRP12 was also recently found to induce inflammasome activation in response to *Y. pestis* infection, and both NLRP3 and NLRP12 contributed to host defense against *Yersinia* infection, presumably via induction of caspase-1-dependent IL-1β and IL-18 (Vladimer et al., 2012). YopJ may activate this NLRP12 inflammasome, although this remains to be demonstrated. Finally, a non-canonical inflammasome pathway involving caspase-11, TRIF, and type I IFN signaling has been described that responds to Gram-negative intracellular bacteria independently of T3SS activity (Kayagaki et al., 2011; Sander et al., 2011; Broz et al., 2012; Rathinam et al., 2012). Whether this pathway contributes to anti-*Yersinia* host defense remains unknown.

## The role of YopJ-induced death *in vivo*

**Figure 2:**
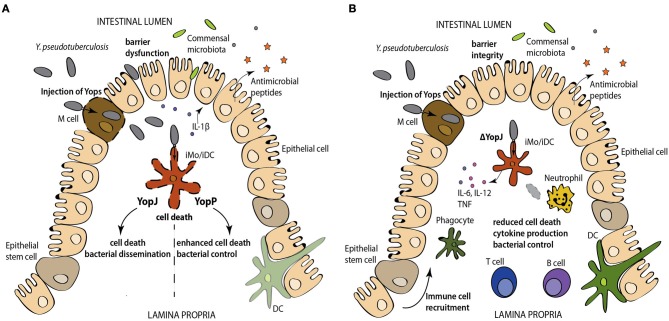
**(A)** Injection of YopJ/P activates caspase-1 and the release of the caspase-1- dependent cytokines. However, the hypercytotoxic YopP-expressing *Y. pseudotuberculosis* induces enhanced levels of cell death and promotes bacterial control, relative to infection with YopJ-expressing *Y. pseudotuberculosis.* **(B)** Conversely infection with YopJ-deficient *Y. pseudotuberculosis* results in robust cytokine production, intact barrier function and control of bacterial spread. Abbreviations: DC, dendritic, iMo/iDC, intestinal macrophage/intestinal DC.

A number of studies indicate that YopJ/P promotes *Yersinia* virulence. Oral infection with *Y. pseudotuberculosis* and *Y. enterocolitica* demonstrate that YopJ/P contributes to systemic disease and barrier dysfunction (Monack et al., 1998; Jung et al., 2012; Meinzer et al., 2012). YopJ is dispensable for colonization of the Peyer's patches (PPs) and mesenteric lymph nodes (mLNs), especially at higher infectious doses; however, YopJ-deficient *Yersinia* had significantly reduced levels of spleen colonization (Monack et al., 1998). Furthermore, spleens and mLNs from mice infected with YopJ-sufficient bacteria had a higher percentage of Mac1^+^ TUNEL^+^ and total TUNEL^+^ cells compared to YopJ-deficient bacteria, consistent with the role of YopJ in apoptosis *in vivo*. Furthermore, in competitive index experiments, YopJ-deficient *Yersinia* showed colonization defects in PPs, mLNs, and spleen. YopJ-deficient *Yersinia* were not defective for splenic replication following intraperitoneal infection, indicating that YopJ primarily regulates dissemination from mucosal tissues, rather replication at systemic sites (Monack et al., 1998) (Figure 2). Consistently, YopJ-deficient *Y. pestis* are still able to cause systemic infection in a rat model of bubonic plague, despite a defect in induction of apoptosis and cytokine inhibition (Lemaitre et al., 2006). These initial findings thus implied that apoptosis may be utilized by *Yersinia* to eliminate immune cells and dampen anti-*Yersinia* immunity during infection of peripheral or mucosal tissues.

Paradoxically, ectopic expression of a hypercytotoxic YopP from *Y. enterocolitica* in *Y. pseudotuberculosis* results in its attenuation in oral mouse infection (Brodsky and Medzhitov, 2008) (Figure 2). While both *Y. pseudotuberculosis* and *Y. enterocolitica* cause cell death in cultured macrophages and infected tissues, infection with YopJ- vs. YopP-expressing *Y. pseudotuberculosis* showed a significant increase in TUNEL^+^ CD11b^+^, CD11c^+^ and B220^+^ cells in mLNs in mice infected with the YopP-expressing strain (Brodsky and Medzhitov, 2008). Similarly, *Y. pestis* strains expressing YopP had higher cytotoxic potency than strains expressing YopJ, both *in vitro* and in tissues of infected mice; furthermore, expression of YopP in *Y. pestis* also resulted in lower virulence following subcutaneous, but not intranasal or intravenous routes of infection (Zauberman et al., 2009). Interestingly, subcutaneous administration of *Y. pestis* expressing YopP protected against infection with virulent *Y. pestis*, regardless of the route of challenge. These observations suggest that YopJ contributes to dissemination of *Yersinia* from barrier surfaces, but may be less important once bacteria have spread to systemic sites. Whether YopJ or additional immunosuppressive virulence mechanisms play a role in dampening the early inflammatory response to *Yersinia* infection in pneumonic plague (Lathem et al., 2007) also remains to be determined.

Consistent with observations that YopJ promotes systemic dissemination following oral infection, YopJ contributes to gut barrier disruption (Jung et al., 2012; Meinzer et al., 2012). Specifically, YopJ can induce TLR2-dependent IL-1β secretion in PPs, which was associated with increased barrier permeability, suggesting that TLR2 signaling mediates YopJ-dependent gut disruption (Jung et al., 2012). However, the actual bacterial loads in these mice were not measured. Conversely, TLR2-deficient mice have been reported to be more susceptible to oral infection by *Y. pseudotuberculosis*, due to a loss of TLR2-dependent Reg3β expression in the gut epithelium (Dessein et al., 2009). Thus, the precise role of TLR2 in *Yersinia* infection remains to be further dissected. Notably, IL-1α is associated with pathological intestinal inflammation and increased dissemination of *Y. enterocolitica* (Dube et al., 2001), but the role of YopP or TLR2 in this context has not been examined.

In contrast to cell death induced by the activity of a bacterial virulence factor, CD8^+^ cytotoxic T cells also induce death of *Yersinia*-infected cells, and are important for control of *Yersinia* infection, as demonstrated by the more severe disease in infected β2m^−/−^, anti-CD8α-treated, or perforin-deficient mice (Bergman et al., 2009). CD8^+^ T cell-mediated killing of bacteria-associated cells targeted them for phagocytosis by uninfected macrophages, and could bypass the anti-phagocytic activity of *Yersinia* Yops (Bergman et al., 2009). Notably, CD8^+^ T cells were not responsible for resistance to YopP-expressing *Y. pestis*, (Zauberman et al., 2009). The more cytotoxic YopP may bypass the requirement for CD8^+^ T cell-mediated killing due to elevated cytotoxicity induced by the bacteria themselves. These studies collectively suggest that regulation of cytotoxicity during *Yersinia* infection impacts virulence, and that a balance between the cytokine-blocking and death-inducing functions of YopJ is required for optimal virulence. Specifically, absence of YopJ results in failure of *Yersinia* to suppress cytokine production or induce cell death and causes a defect in dissemination. However, *Y. pseudotuberculosis* expressing YopP, which enables stronger inhibition of cytokine production and elevated levels of cell death, are also significantly attenuated *in vivo* (Figure 2). Thus, while the relative contributions of bacteria-induced and T cell-induced cell death during *Yersinia* infection *in vivo* are not yet defined, activation of cell death *in vivo* either in response to YopJ activity, or as a consequence of T-cell-mediated cytotoxicity likely promotes immune responses against *Yersinia*.

## *In vivo* consequences of cell death during infection

Apoptotic cell death is generally viewed as non-inflammatory or immunosuppressive, and YopJ-dependent cell death has been characterized as apoptosis (Mills et al., 1997; Monack et al., 1997, 1998; Ruckdeschel et al., 1997, 1998). However, apoptotic cell death can also promote immune responses. For example, anthracyclin treatment of tumor cells causes apoptosis that leads to exposure of calreticulin on the surface, which acts as a signal to induce phagocytosis and promote anti-tumor T cell responses *in vivo* (Kepp et al., 2009). Similarly, the dendritic cell C-type lectin receptor, DNGR-1, can promote cross-presentation of apoptotic cell antigens by CD8α^+^ DCs to CD8^+^ T cells in both non-infectious and infectious settings (Sancho et al., 2009; Ahrens et al., 2012). Dendritic cells that phagocytose bacterially-infected apoptotic cells produce both the immunoregulatory cytokine TGFβ, and the inflammatory cytokine IL-6, which together promote the differentiation of naïve CD4^+^ T cells into T_H_17 cells (Torchinsky et al., 2009). These cells play a critical role in anti-bacterial immunity and pathological inflammatory responses at mucosal barrier surfaces (Ye et al., 2001; O'Connor et al., 2009; Sonnenberg et al., 2010). Investigating whether these mechanisms promote immune responses against *Yersinia* infection may provide new insights into both anti-*Yersinia* immunity and potential bacterial evasion strategies (Lin et al., 2011; Smiley, 2008).

## Concluding remarks

Understanding the control of programmed cell death remains essential for understanding anti-bacterial immunity. Appropriately distinguishing pathogens and non-pathogens is critical both for host defense and the maintenance of tissue homeostasis. The ability to detect conserved bacterial virulence activities, such as the disruption of actin cytoskeleton or inhibition of core signaling pathways, provides a mechanism for rapidly sensing the presence of pathogens. Induction of cell death in response to microbial virulence activities is an evolutionarily conserved response from plants to higher eukaryotes that limits the spread of infection. In higher organisms, distinct forms of programmed cell death, including apoptosis, pyroptosis, and necrosis, can have differential effects on downstream responses. Various cell types may have differing thresholds for activation of these distinct death pathways due to differential expression of pro-survival, pro-apoptotic, and immune sensor proteins. Thus, understanding how cells die and how cell death influences both the local microenvironment and ensuing systemic response may provide key insight into new approaches to modulate both antimicrobial immune responses and immunopathologies that result from dysregulation of these death pathways.

### Conflict of interest statement

The authors declare that the research was conducted in the absence of any commercial or financial relationships that could be construed as a potential conflict of interest.
